# Painful Legs and Moving Toes Syndrome: Case Report and Review

**DOI:** 10.3390/neurolint16060102

**Published:** 2024-11-04

**Authors:** Mihael Tsalta-Mladenov, Vladina Dimitrova, Silva Andonova

**Affiliations:** 1 Second Clinic of Neurology with ICU and Stroke Unit, University Hospital “St. Marina”, 9000 Varna, Bulgaria; 2 Department of Neurology and Neuroscience, Faculty of Medicine, Medical University “Prof. Paraskev Stoyanov”, 9000 Varna, Bulgaria; 3 Department of Optometry and Occupational Diseases, Faculty of Public Health, Medical University “Prof. Paraskev Stoyanov”, 9000 Varna, Bulgaria

**Keywords:** painful legs and moving toes syndrome, PLMT, Movement Disorder, restless legs syndrome, pain, painful legs, moving toes, involuntary, Pregabalin, spinal cord stimulation, SCS, botulinum

## Abstract

Introduction: Painful legs and moving toes (PLMT) syndrome is a rare movement disorder characterized by defuse lower limb neuropathic pain and spontaneous abnormal, involuntary toe movements. Objective: The objective was to present a rare case of PLMT syndrome with a triggering area in an adult patient due to multilevel discogenic pathology, to make a thorough review of this disorder and to provide a practical approach to its management. Case presentation: A 59-years-old male was admitted to the neurology ward with symptoms of defuse pain in the lower-back and the right leg accompanied by involuntary movements for the right toes intensified by tactile stimulation in the right upper thigh. Magnetic resonance imaging (MRI) revealed a multilevel discogenic pathology of the lumbar and cervical spine, with myelopathy at C5-C7 level. A medication with Pregabalin 300 mg/daily significantly improved both the abnormal toe movements and the leg pain. The clinical effect was constant during the 90-day follow-up without any adverse effects. Conclusion: Painful legs and moving toes (PLMT) is a condition that greatly affects the quality of life of patients, but which still remains less known by clinicians. Spontaneous resolution is rare, and oral medications are the first-line treatment. Pregabalin is a safe and effective treatment option for PLMT that should be considered early for the management of this condition. Other medication interventions, such as botulinum toxin injections, spinal blockade, or non-pharmacological treatment options like spinal cord stimulation, and surgical decompressions, are also recommended when the conservative treatment is ineffective in well-selected patients.

## 1. Introduction

Painful legs and moving toes (PLMT) syndrome is a rare movement disorder characterized by discomfort and defuse pain sensation in the lower extremities and involuntary irregular movements of single or multiple toes [1]. Owing to its rarity, case reports have accounted for the majority of PLMT’s descriptions, and its precise incidence is still unknown [2]. The etiopathogenesis in unclear as most of the cases are due to peripheral nervous system (PNS) disorders such as spinal cord compression, peripheral neuropathy, radiculopathy, and history of trauma [3]. However, it is hypothesized that a crucial part in the development of this syndrome is played by functional alterations in the afferentation of the nociceptive impulses, spinal cord interneurons, sympathetic nervous system, and subsequent reorganizations of the central nervous system (CNS) at the spinal cord level or higher levels [4]. Several therapy modalities, most of which have a temporary effect, have been proposed to manage PLMT syndrome. These include oral medicines, local nerve blocks, injections of botulinum toxin type A, and epidural spinal cord stimulation [3]. In this case report we describe a single patient with PLMT syndrome who had a remote triggering point and who experienced a significant effect regarding both major symptoms—pain and the abnormal toe movements from a low dose anticonvulsant (pregabalin). We also provide an overview of the relevant literature.

## 2. Case Presentation

A 49-year-old male patient presented to the Second Clinic of Neurology at the University Hospital “St. Marina”, Varna, Bulgaria, with complaints lasting for one year of lower back pain. During this period, the pain was radiating toward the dorsolateral region of the right thigh. After six months, the pain drastically changed its characteristic—it became defuse and interfered with his daily activities rated as 8/10 on a visual analogue scale (VAS). He observed involuntary movements for the toes on the right foot—first only the big toe, and later for all the toes. The patient also had a feeling of “numbness” for both thighs, more severe on the right. The patient observed worsening of the involuntary movements when he touched and performed a circular movement on the proximal part of the right thigh. The involuntary movements intensified when the pain was stronger and disappeared during sleep, according to his wife. Three months after a respiratory infection the patient also complained of cervical pain, with irradiation to the left arm toward IV-V fingers. The patient was treated with analgesics, non-steroidal anti-inflammatory drugs (NSAIDs), vitamins from group B, and galantamine—without any improvement of the symptoms.

The patient did not have any chronic diseases, and he did not take any other medication except for the pain relief. He had no previous history of trauma, spinal injuries, or taking antidepressants or antipsychotics and he had no family history of similar complaints or diagnosed movement disorders.

The neurological examination on admission revealed persistent, mild, involuntary movements of the toes of the right foot comprising flexion/extension and abduction/adduction to a lesser extent. The movements were intensified in amplitude and speed and accompanied by pain when the patient performed a circular movement on the proximal part of the right thigh (Video S1). Mechanical skin irritation in other areas of the right leg also provoked involuntary movements, but to a lesser extent.

The patient demonstrated spontaneous involuntary movement consisting of abduction/adduction and twisting movements of the right toes, as well as mild flexion/extension movement of the right ankle, accompanied by pain. These abnormal movements were increased by sensory stimulation (circular movements on the proximal right thigh) and diminished after the stimulation was discontinued.

The abnormal movements, in this case were distinct from the symptom of restless legs syndrome (RLS), as they appeared involuntary, were not accompanied by an irresistible urge to move, and did not fluctuate within the day.

The initial laboratory tests, including blood and urine analysis, were unremarkable.

**Figure 1:**
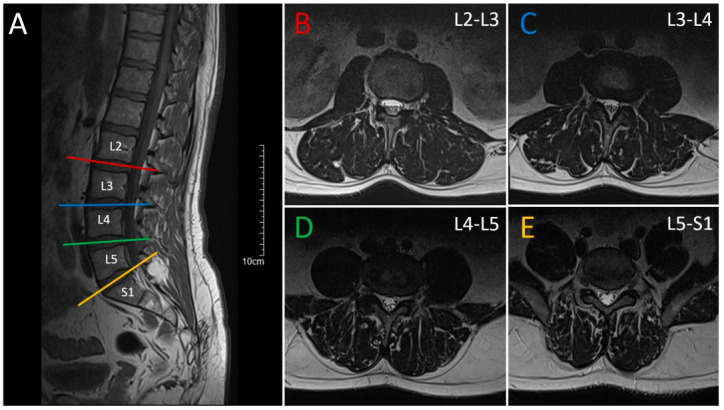
Magnetic Resonance Imaging (MRI) of the lumbar spine. (**A**): T1-weighted sagittal MRI showing spinal cord, degenerative changes in the lumbar spine with multilevel bulging of the intervertebral discs, osteochondrosis and lipoma at the level of L5-S1. Horizontal lines indicate the corresponding levels in the axial views. (**B**–**E**): T2-weighted axial MRI at different levels presenting the degenerative changes and intervertebral disc bulgings C6-C7—median disc herniation with compression of the dural sac and the spinal cord with myelopathy.

An MRI of the lumbar spine revealed a lipoma at the level of L5-S1 and multilevel discogenic pathology with bulging of the intervertebral disc at the levels of L2-L5 (Figure 1).

**Figure 2:**
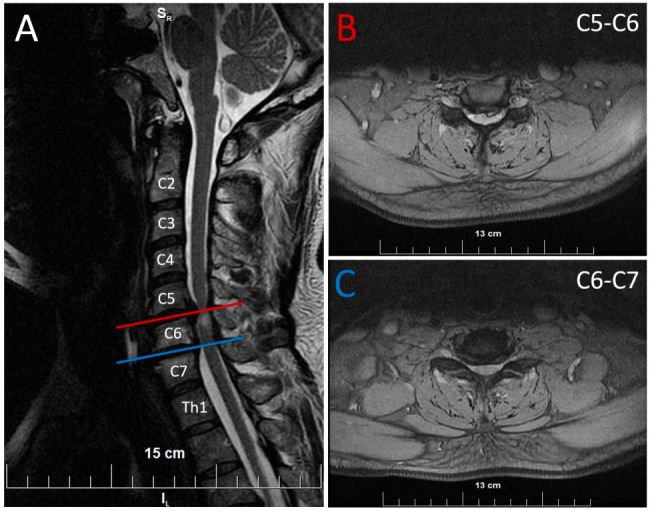
Magnetic resonance imaging (MRI) of the cervical spine. (**A**): T2-weighted sagittal MRI showing spinal cord, bulging of the intervertebral discs at all cervical levels and disc herniation at levels C5-C7 with myelopathy. Horizontal lines indicate the corresponding levels in the axial views. (**B**): T2-weighted axial MRI at the level of C5-C6 — paramedian disc herniation to the left with compression of the dural sac and the spinal cord with myelopathy. (**C**): T2-weighted axial MRI at the level of C6-C7—median disc herniation with compression of the dural sac and the spinal cord with myelopathy.

The MRI of the cervical spine revealed degenerative changes with bulging of the disc at C3-C4 and a disc herniation at level C5-C7 with myelopathy (Figure 2).

The EMG revealed decreased amplitudes from the compound muscle action potential (CMAP) and mild slowing of conduction velocity for n. peroneus bilaterally and n. medianus on the right indicative for mixed peripheral neuropathy with axonal degeneration and demyelination. Also, EMG criteria for cervical (C5-C7) and lumbar (L4-S1) radiculopathy were met.

Based on the clinical characteristics of the involuntary movements for the right toes with excruciating pain and the MRI findings, a diagnosis of PLMT due to multilevel discogenic pathology was made. It was discussed that the presence of spontaneous involuntary toe movements and their intensification upon tactile stimulus on the right proximal thigh might be due to the formation of a triggering point. We propose that the efferent motor fibers are the target of a considerable over-threshold interneuron simulation caused by the summed afferent sensory stimuli, which ultimately intensifies the local pain syndrome and the abnormal movements.

After consultation with a neurosurgeon, the patient was assessed as a good candidate for decompression surgery of the cervical spine due to the disk herniations C5-C7 with myelopathy, but the patient refused a surgical treatment. Therefore, a decision for oral treatment with Pregabalin was made with a gradual titration of the drug up to 300 mg/daily in two intakes. Upon reaching the therapeutic dosage, the patient reported marked reduction of the pain syndrome, and also of the involuntary toe movements up to total disappearance of them. At the 90 days follow-up period there was a good compliance with treatment, which was still effective with no adverse effects. There were no spontaneous nor provoked involuntary movements, but mild pain syndrome was still present, rated as 2–3/10 on a visual analogue scale (VAS).

## 3. Review

### 3.1. Historical Data

Painful legs and moving toes syndrome (PLMT) is a rare neurological movement disorder. The first report of PLMT was in 1971 by the Welsh neurologist John David Spillane and his colleagues [1]. His description was very detailed, presenting the pain as deep aching pulling in the feet or lower limbs with spontaneous movements of the toes. The movements are always localized to the toes and more rarely to the feet and the more proximal muscles of the limbs. These spontaneous and purposeless movements include flexion and extension, and adduction and abduction of the toes combine to cause a continual wriggling and writhing movement [1]. Later, in 1985, after a description of cases affecting the upper limbs, the definition was expanded to the upper limbs: painful hands and moving fingers (PHMF) [5]. The affected locations have extended from toes or fingers proximally to ipsilateral or contralateral limbs in some cases, which was the reason to call this syndrome “painful limbs and moving extremities” (PLME) [6]. Additionally, there are a few cases of the painless variant of PLMT, that are raising further concerns about the classification of the condition [7].

Currently, single case and case series are rarely being reported in the literature, as currently there are 143 reports of PLMT, according to our literature search on PubMed and other public open-access databases (screening on 1 July 2024).

### 3.2. Etiopathogenesis

#### 3.2.1. Etiology

Even after five decades, the etiopathogenesis of PLMT is currently unclear. There are numerous theories and possible mechanisms of the syndrome discussed in relation to the previously reported cases [3]. The most common of them include neuropathological conditions mostly in peripheral, and more rarely in central, nerves, like post-traumatic peripheral nerve injury, focal nerve injury, polyneuropathy, restless leg syndrome (RLS) or injuries of the spinal cord, cauda equina and the nerve roots, as well as postoperative complications [8,9,10,11,12]. In more rare cases other neurological conditions have been considered to be related to PLMT, such as stroke, Parkinson’s Disease, Wilson’s Disease, as well as some viral infections affecting the peripheral nervous system such as the varicella-zoster virus (VZV). The most common conditions associated with PLMT are presented in Table 1.

#### 3.2.2. Pathogenetic Mechanisms

**Figure 3:**
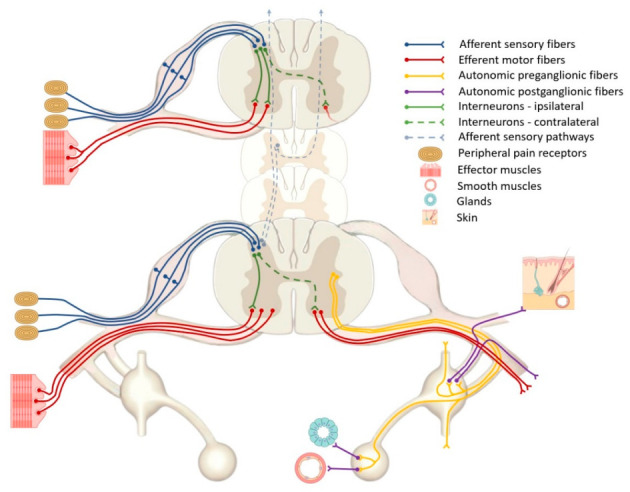
Leading pathogenetic mechanisms of painful legs and moving toes syndrome (PLMT)—role of interneurons, autonomic fibers, and remote trigger points.

Even after a thorough examination of about 40% of the cases, the etiology could be identified [3]. After numerous clinical observations it was assumed that the involuntary toe movements are due to abnormally activated neurons in the anterior horn of the spinal cord due to the constant pathological firing of impulses from afferent fibers of an injured nerve of the sympathetic nervous system [3]. The theory that the sympathetic neuronal signals could provoke pain and unvoluntary movements by activating a so-called “impulse generator” at the injured location of affected limbs was proven by improvement of the symptoms in a case-series treated with sympathectomy [13]. Further investigations were focused on the pain pathway [3]. In general, there is an activation of the nociceptors at the site of the nerve injury (transduction), transferring the neuronal signals from peripheral neurons to central ones in the dorsal horn of the spinal cord, brain stem, and the thalamus (conduction and transmission). There is also inhibition in the spinal cord, activation of descending inhibitory tracts (modulation), and projection to the somatosensory cortex, enabling the perception of pain (perception) [14]. Spinal cord interneurons may partially transmit abnormal neural signals from the afferent sensory structures to segmental or multisegmental efferent motor pathways, which may reproduce simple or more complex abnormal involuntary movements (Figure 3) [15].

Chronic primary pain, such as neuroplastic pain, can be part of the pathogenesis of chronic pain over time, due to central pain modulating disturbances, including three major mechanisms: increased excitability of ascending and descending facilitatory tracts, reduced inhibition of descending anti-nociceptive tracts, and psychogenic conditions. These facts are also important for further chronic pain management [14,16]. In most cases, PLMT could begin with peripheral nervous system (PNS) causes and be associated later with subsequent changes in central nervous system (CNS) [6]. Frequent ectopic impulses to the posterior root, which are spontaneously produced at the injured peripheral nerve, could lead to pain and involuntary movements via the abnormal re-networking of local spinal circuits [15].

The involuntary movements become more severe when the pain becomes worse. Pain-exacerbating or improving features are often identified in about 60% of the cases, including various body positions or activities such as sitting, walking, or bending. There are also differences in the symptom severity due to diurnal variations, cold temperature, and external pressure [16]. The mechanoreceptive nociceptors typically react to strong, possibly damaging mechanical stimuli, and they have a high threshold for activation [17]. A trigger point may arise as a result of several subthreshold sensory stimuli when the sum of their potentials exceeds a particular threshold. Usually trigger points are located in the level of the pathology, or in rare cases remotely from it. Afferent impulses may cause aberrant interneuron simulation in response to above-threshold stimulation, which can manifest clinically as movement amplification.

### 3.3. Clinical Workout

#### 3.3.1. Diagnosis

The diagnostics of PLMT are based on a thorough medical history and detailed clinical, neurophysiological, and neuroimaging examination [18]. First, the medical history is not specific and can reveal only possible pathological conditions related with PLMT, such as those presented in Table 1. Second and most important is the clinical assessment of the patient as the clinical workflow is in two major directions, namely, the pain syndrome and the involuntary movements [3].

In the majority of the documented cases, discomfort and defuse pain in the lower limbs are the first complaints [11]. Typically the pain is neuropathic, felt deep in the affected limbs, not following the classic body distributions such as segmental, peripheral nerve, or distal type [19]. Nevertheless, the description of the pain can vary in very large ranges from bursting, tearing, tingling, only numbness, cramp-like, aching, electric sensation, and pulling, etc. [3]. The diverse descriptions of pain are probably due to the different underlying conditions and both sensory and affective disturbances due to the prolonged neuropathic pain.

The involuntary movements of the toes are in various planes, namely, flexion/extension, adduction/abduction, or a combination of them. In most cases, there is a movement of one or more toes, typically unilaterally, but in some cases, there might be a bilateral symptom [18]. The movements are mostly irregular, described as dystonic or myoclonic, and more rarely they can be rhythmic [10]. Case-specific and over time, the movements can spread and affect more fingers and larger areas such as ipsilateral proximal muscle groups and even the contralateral limb [20]. Intensification of the movements might be observed if the pain syndrome is aggravated [18] and also at rest at night before sleep [11]. The involuntary movements disappear during sleep, which can be due to the inhibition of the brainstem reticular system [3]. Normally, the movements cannot be voluntarily inhibited, whereas there are some single cases of brief voluntary inhibition for seconds [3,11,18,21]. It is recommendable to videotape the involuntary movements in rest and also during activation, if a triggering point is present, in order to maintain a profile of the condition’s dynamics over time.

Both electroneurography (ENG) and electromyography (EMG) are crucial neurophysiological examinations able to reveal a possible underlying condition such as radiculopathy or peripheral nerve damage conditions resulting in PLMT syndrome. During EMG, spontaneous irregular bursts can be recorded. Due to this, ENG with segmental stimulation can reveal the site of the lesion and its severity [18].

The choice of neuroimaging techniques is case-dependent, but in most cases the investigations are focused on the spinal cord and the spinal column. They can give additional information about the underlying conditions associated with the development of PLMT. Magnetic resonance imaging (MRI) is the “gold standard” for morphological investigation. Whenever MRI is not available, Computed tomography (CT) might be used instead. With these imaging modalities, we can rule out injuries or the presence of structural abnormalities or other conditions, such as: tumors, disc herniations, degenerative changes of the spinal column, inflammation of spinal cord, and others.

#### 3.3.2. Differential Diagnosis

Based on all the clinical findings and investigations a possible painful leg and moving toes (PLMT) syndrome case must be differentiated from other neurological diseases (Table 2):

### 3.4. Treatment Options

The treatment of PLMT is challenging as there are no guidelines or consensus of recommendations for the management of this condition [11,18]. The current approach is symptomatic and case-based, focused on the individual’s possible etiopathogenesis and the leading symptom of either the pain syndrome or the involuntary movements [3,22]. There are no differences in the treatment response in symptomatic or cryptogenic patients [23]. The persistence of chronic pain syndrome in many cases leads to a central remodeling and hardwiring of pain, making the syndrome very difficult to improve [24].

Different pharmacological and non-pharmacological therapies are being reported to be effective in single PLMT cases, as the treatment is mostly for the pain syndrome because in the majority of the cases this is the dominant complaint [8,11,18,25].

#### 3.4.1. Pharmacological Treatment

Oral medications are often preferred as a first-line treatment.

The most commonly used medications are modulators of the gamma-aminobutyric acid (GABA), which is a major inhibitory neurotransmitter, and plays pivotal roles in the regulation of pain sensation. GABA-ergic, such as pregabalin [8,11] and gabapentin [10,26,27,28,29], are being reported to have a satisfying effect on pain reduction, improvement of quality of life and even sleep disturbances in adult and pediatric PLMT patients. Unfortunately, some cases describe a significant initial effect, but during long-term follow up there is a reduction of the effect, probably due to downregulation of the GABA receptors and more rarely due to discontinuation of the treatment due to drug-specific side effects such as dizziness and leg edema [11]. Nevertheless, GABA-ergic drugs should be considered as an early treatment option due to their proven effect on chronic pain syndromes and good safety profile.

Other oral medications, such as analgesics [30], corticosteroids [30,31], myorelaxants (baclofen [32,33]), beta-blockers (propranolol [12,30]), benzodiazepines ( [30,33,34,35]), anticonvulsants (carbamazepine [27,32,36,37]), tricyclic antidepressants (amitriptyline [30,33]), atypical antipsychotics (quetiapine [8,38]), and non-ergoline dopamine agonist (pramipexole [39]) are also being reported to have individually transient improvement of the symptoms, but without a satisfactory outcome. These drugs can be recommended as a monotherapy, as well as a combination, depending on the suspected etiopathogenesis of PLMT and the clinical outcome.

Various medication interventions could be considered in severe pain PLMT cases.

Epidural blockages with locally injected anesthetics have a good temporary improvement of pain even in cases with herpes zoster associated myelitis [3,40].

Lumbar sympathetic blockade is being considered as a treatment option ever since the very first report of it in 1971 [1]. Several cases in the literature are proving the benefit of the sympathetic blockade and in some of them, even a full disappearance of the symptoms is reported [41,42]. Unfortunately, the duration of the effect is not being specified and, in most cases, it is brief and transient.

Recently there is a growing number of cases treated with local intramuscular injections of Botulinum neurotoxin A (BoNT-A). The rising interest in this treatment option is based on the fact that it can relieve both the pain syndrome and the involuntary movements. BoNT-A is a nerve blocker at the neuromuscular conjunction, that leads to improvement of the involuntary movements, but also to pain reduction via blockage of the exocytosis of different inflammatory mediators related to pain sensation (substance P, glutamate, calcitonin gene-related peptide (CGRP)) [43,44]. Administration of BoNT-A is recommended as monotherapy or in combination with other treatments in PLMT cases where the oral medication and other therapeutic intervention did not show a sufficient effect [45,46]. The effect duration is about 10–12 weeks, and it is sometimes shorter, as well as longer. This treatment is recommended as a safe and effective symptomatic treatment in moderate to severe symptoms of PLMT with a mean effect duration of 10–12 weeks. After this period, a re-administration should be considered. In rare cases after a long-term treatment, the formation of neutralizing antibodies can lead to a lack of clinical effects and secondary non-responders [47].

#### 3.4.2. Non-Pharmacological Treatment

Spinal cord stimulation (SCS) is a minimally invasive neurosurgical method, in which a temporary and later a permanent “pacemaker”-like device is implanted in the abdominal area and a epidural stimulating electrode is implanted. SCS has been proven as highly effective treatment option for various pain syndromes such as complex regional pain syndrome, back pain, failed-back surgery (FBS) syndrome, and even peripheral vascular disease and diabetic polyneuropathy. In the last two decades a few cases have been treated with epidural SCS, as the initial results have been promising [3]. Improvement in both pain syndrome and the involuntary movements up to complete resolution of the toe movements have been achieved via SCS [48,49]. The effect is reported to be constant over time up to 1 year after the initial neuromodulation, and probably even longer due to the option to adjust the parameters of neuromodulation over time [48,49]. Nevertheless, a good patient selection is required for SCS treatment, as some of the patients, such as those with herpes zoster myelitis, will have no benefit from it [40].

Sympathectomy is being reported as a beneficial surgical option in single cases [13]

Decompressive neurosurgery is recommended in cases with compression of different nerve structures ranging from radiculopathy to myelopathy. In multiple cases, such etiology was associated with development of PLMT syndrome, and nerve decompression results demonstrated significant reduction up to complete disappearance of the pain and involuntary movements [5,15].

Different physical methods, such as transcutaneous electrical nerve stimulation (TENS), local vibration, heat or cold therapy, and tactile stimulation (sensory tricks and maneuvers), can be used for temporary pain relief, but without significant long-term effects [3,50].

## 4. Conclusions

We reported an adult case of PMLT syndrome in which the leg pain and involuntary toe movements were significantly relieved by pregabalin. Although the exact etiopathogenesis of PLMT is unknown, PNS disease is typically associated with this illness. The quality of life of patients is greatly affected by this uncommon syndrome, while clinicians are not well aware of it. The therapeutic approach is individually tailored as oral medications are the first line treatment. Pregabalin is widely used for neuropathic pain management and is also effective for PLMT patients and should be early considered for this condition. Therapeutic interventions such as BoNT-A injections, spinal blockade, spinal cord stimulation, and surgical decompressions are also recommended when the conservative treatment is ineffective in well-selected patients.

## Figures and Tables

**Table 1:** Conditions associated with the development of PLMT.

Conditions Associated with Painful Legs and Moving Toes (PLMT) Syndrome		
Peripheral Nervous System (PNS) Disorders	Central Nervous System (CNS) Disorders	Non-Neurological Conditions
Spinal cord compression	Stroke	Hashimoto thyroiditis
Lumbar canal stenosis	Parkinson’s Disease	Treatment with neuroleptics
Disc herniation	Wilson’s Disease	
Spinal tumors	Neuroinfections—myelitis (Varicella-zoster virus associated)	Dupuytren’s contracture
Tethered cord syndrome		
Polyneuropathy		
Radiculopathy		
Plexus injury		
Cauda equina trauma		
Local trauma without documented neurological injury		
Postsurgical complication		

**Table 2:** Differential diagnosis of painful legs and moving toes (PLMT) syndrome.

Differential Diagnosis of Painful Legs and Moving Toes (PLMT) Syndrome	
Conditions Presenting with Chronic Pain Syndrome	Conditions Presenting with Involuntary Movements
Radiculopathy	Restless leg syndrome (RLS)
Plexopathy	Chorea
Polyneuropathy	Myoclonus
Disc herniation	Dystonia
Spinal cord stenosis	Parkinson’s Disease (PD)
Spinal tumor	Akathisia with leg involvement
Spinal injury	Periodic-limb-movement disorder (PLMD)
Cramps	Epilepsia partialis continua (EPC)
	Functional movement disorders (FMD)

## Data Availability

The original contributions presented in the study are included in the article, further inquiries can be directed to the corresponding author.

## Supplementary Materials

The following supporting information can be downloaded at: https://www.mdpi.com/article/10.3390/neurolint16060102/s1, Video S1: Painful legs and moving toes (PLMT) syndrome: Involuntary movements of the right toes before treatment.
